# Comparison of continuous infusion and intermittent boluses of furosemide in acute heart failure: A protocol for systematic review and meta-analysis

**DOI:** 10.1097/MD.0000000000027108

**Published:** 2021-09-17

**Authors:** Tao Wang, Yue Han, Yu Song

**Affiliations:** Department of Cardiology, Fuyang Cancer Hospital, Fuyang, Anhui, China.

**Keywords:** continuous, furosemide, heart failure, intermittent, meta-analysis

## Abstract

**Background:**

Acute heart failure (HF) is a common cause of hospital admission. This study aims to compare continuous infusion and intermittent boluses of furosemide in treating acute HF.

**Methods:**

This protocol of systematic review and meta-analysis has been drafted under the guidance of the preferred reporting items for systematic reviews and meta-analyses protocols. Electronic databases including Web of Science, Embase, PubMed, Wanfang, Data, Scopus, Science Direct, and Cochrane Library will be searched in June 2021 by 2 independent reviewers. The main outcomes are post-treatment daily urine output, weight, length of stay, serum sodium, potassium, and creatinine. Two researchers conducted a quality assessment in strict accordance with the risk bias assessment tool recommended by the Cochrane Handbook Version5.3. We performed the meta-analysis by Stata version 10.0 software.

**Results:**

The results of this systematic review and meta-analysis will be published in a peer-reviewed journal.

**Conclusion:**

The choice of furosemide regime in acute HF remains physician preference. Both bolus and continuous infusion yields satisfactory outcomes.

## 1 Introduction

Acute heart failure (HF) is common and potentially fatal.^[1,2]^ With an estimated global prevalence of 2% in the adult population, it is a significant burden on healthcare systems globally.^[3]^ Multiple factors may exacerbate HF, commonly being myocardial ischaemia/infarction and infections.^[4,5]^ Diuretics, are the backbone of acute HF therapy because they facilitate intense urine output shortly after administration.^[6,7]^ Despite loop diuretics having been used for several decades, there is still a large variation in the strategies used to administer these drugs. In terms of clinical practice, most physicians prefer to give intravenous loop diuretics in repeated boluses because this schedule is easier to perform than continuous infusion.^[8]^ However, boluses of loop diuretics may lead to high peak plasma drug levels and abrupt changes in intravascular volume, which can lead to renal toxicity.^[9]^ In addition, this therapeutic regimen may be associated with a drug-tolerance effect due to compensatory sodium retention once the action of the drug has finished. In this regard, some studies have demonstrated that continuous infusion diminishes fluctuations in intravascular volume and is accompanied by a constant urine production over time and also prevents high plasma peaks of the drug which, in turn, limits the risk of toxicity.^[10]^

While the European Society of Cardiology specifically recommended using the smallest dose of furosemide as adjusted according to renal function to bring about clinical effects in acute HF,^[11]^ citing primarily the Diuretic Optimization Strategies Evaluation (DOSE) as evidence,^[12]^ both intermittent boluses or continuous infusion were said to be acceptable. Nonetheless, such details in regimen may be important clinically and may alter outcomes. As such, this study aimed to summarize the existing evidence comparing continuous infusion and intermittent boluses of furosemide in acute HF, evaluating in particular the efficacy of diuresis, safety, and clinical outcomes of the 2 regimens.

## 2 Methods

### 2.1 Protocol registration

The prospective registration has been approved by the Open Science Framework registries, and the registration number is 10.17605/OSF.IO/P47D3. The protocol was written following the Preferred Reporting Items for Systematic Reviews and Meta-Analyses Protocols (PRISMA-P) statement guidelines.^[13]^ Ethical approval and patient consent are not required because this study is a literature-based study.

### 2.2 Searching strategy

Electronic databases including Web of Science, Embase, PubMed, Wanfang, Data, Scopus, Science Direct, and Cochrane Library will be searched in June 2021 by 2 independent reviewers. The search string used is acute AND “heart failure” AND (diuresis OR frusemide OR furosemide OR lasix OR diuretic OR loop). To minimize the risk of publication bias, we will conduct a comprehensive search that included strategies to find published and unpublished studies. The reference lists of the included studies will also be checked for additional studies that are not identified with the database search. There is no restriction in the date of publication or language in the search.

### 2.3 Inclusion and exclusion criteria

Two independent researchers removed duplicated articles by using EndNote and then screened the titles and abstracts of articles to exclude irrelevant studies. Then they reviewed the full-texts of the remaining records independently to determine eligibility for this meta-analysis according to following inclusion criteria: randomized controlled trials (RCTs) included crossover, cluster, and quasi-RCT designs; the objects were definitely diagnosed with acute HF, and their ages were over 18 years old; the intervention group received continuous infusion of furosemide, while the control group received intermittent boluses of furosemide; the studies included one of the following indicators: post-treatment daily urine output, weight, length of stay, serum sodium, potassium, and creatinine. Any inconsistencies were resolved by consulting the third researcher.

## 3 Data extraction

Two reviewers will be responsible for information extraction according to the following information: the basic information of the included studies, including the first author, the year of publication, etc. The basic characteristics of the subjects, including the number of patients in the treatment group and the control group, sex composition, average age, intervention drug dosage, treatment course, and other specific details.

### 3.1 Evaluation of bias

Two researchers conducted a quality assessment in strict accordance with the risk bias assessment tool recommended by the Cochrane Handbook Version5.3. Topics include: randomization, allocation concealment, blinding of researchers and subjects, blind evaluation of study results, the integrity of outcome data, selective reporting bias, and other biases. The quality of the literature was rated as “high risk,” “low risk,” and “unclear risk.”

Quality of evidence was appraised with the Grading of Recommendation Assessment (GRADE) method including the risk of bias, inconsistency, indirectness, imprecision, and publication bias by the GRADEpro GDT 2015 to create SoF table. Disagreements over the risk of bias in particular studies will be resolved by discussion, which routinely implicated a third researcher if necessary.

## 4 Statistical analysis

We performed the meta-analysis by Stata version 10.0 software (The Cochrane Collaboration, Oxford, United Kingdom). and calculated the statistics using the inverse variance statistical method. Continuous variables were expressed as the weighted mean difference (WMD) or standardized mean difference (SMD) and 95% confidence interval (CI). WMD was used when data were measured in the same scale and SMD were used if data were measured using different scales. Heterogeneity among the studies was quantified with the *I*^2^ statistic. If *I*^2^ > 50% or *P* < .1, a random effect model was used to decrease heterogeneity, and the subgroup and sensitivity analysis were performed to explore the sources of heterogeneity; otherwise, heterogeneity was negligible and a fixed-effect model was used. Publication bias was assessed using the Egger linear regression test, and *P* < .05 indicated no publication bias among the included studies.

## 5 Discussion

Acute HF is a common cause of presentation to the emergency department and hospital admission.^[14,15]^ Intravenous diuretics, in particular loop diuretics such as furosemide, have long been established as a core pharmacotherapy for acute HF patients in general, even though the 2013 American guideline only gave an evidence level of B (evidence from a single randomized controlled trial/non-randomized studies). The choice between continuous infusion and intermittent boluses was left to physicians’ preference by both guidelines. Therefore, it is necessary to formulate this systematic review and meta-analysis to synthesize these accessible clinical evidence, and we hope this systematic review will provide more comprehensive, reliable, and practical evidence for clinical decision-making and further research. The existing studies on this subject generally have small sample sizes and are mostly cross-over trials for which concern regarding baseline characteristics of the participants have been raised. Further prospective studies involving larger patient populations and longer follow-up periods may provide us with a stronger evidence in the future to more adequately answer questions on this issue.

## Author contributions

**Conceptualization:** Yue Han.

**Data curation:** Yue Han.

**Formal analysis:** Yu Song, Yue Han.

**Funding acquisition:** Tao Wang.

**Investigation:** Yu Song.

**Methodology:** Yu Song.

**Writing – original draft:** Tao Wang.
